# Z-Ajoene Inhibits Growth of Colon Cancer by Promotion of CK1α Dependent β-Catenin Phosphorylation

**DOI:** 10.3390/molecules25030703

**Published:** 2020-02-06

**Authors:** Hua Li, Ji Hye Jeong, Sung Won Kwon, Sang Kook Lee, Hwa Jin Lee, Jae-Ha Ryu

**Affiliations:** 1 Research Institute of Pharmaceutical Sciences and College of Pharmacy, Sookmyung Women’s University, Seoul 04310, Korea; cooldog227@hotmail.com (H.L.); jjh4415@naver.com (J.H.J.); 2 College of Pharmacy, Seoul National University, Seoul 08826, Korea; swkwon@snu.ac.kr (S.W.K.); sklee61@snu.ac.kr (S.K.L.); 3 School of Industrial Bio-Pharmaceutical Science, Semyung University, Jecheon 27136, Korea

**Keywords:** colon cancer, β-catenin, Z-ajoene, phosphorylation, casein kinase 1α

## Abstract

Aberrant activation of a Wnt/β-catenin pathway results in nuclear accumulation of β-catenin in colon cancer. Inhibiting β-catenin is one strategy for treating colon cancer. Here, we identified Z-ajoene, a sulfur containing compound isolated from crushed garlic, as an inhibitor of colon cancer cell growth. Z-Ajoene repressed β-catenin response transcriptional activity, intracellular β-catenin levels, and its representative target protein levels (c-Myc and cyclin D1) in SW480 colon cancer cells. To clarify the regulatory mechanism of decreased β-catenin levels, we examined the effect of Z-ajoene on β-catenin phosphorylation, which is involved in β-catenin degradation. Z-Ajoene promoted the phosphorylation of β-catenin at Ser45 in a casein kinase 1α (CK1α)-dependent manner, which is an essential step in β-catenin degradation in the cytosol. These findings indicate that Z-ajoene from garlic may be a potential chemotherapeutic agent by modulating CK1α activity and the Wnt/β-catenin signaling pathway.

## 1. Introduction

Colon cancer is a common cancer associated with high incidence and high mortality [1]. About 75% of colon cancers arise from truncation in the adenomatous polyposis coli (APC) gene, resulting in deregulation of the Wnt signaling and the accumulation of cytosolic β-catenin [2,3]. β-Catenin, a co-transcription regulator of physiological homeostasis, is a critical regulator of Wnt signaling. Without a Wnt ligand, β-catenin is degraded in the cytoplasm by destruction complex machinery, including axin, adenomatosis polyposis coli (APC), glycogen synthase kinase 3 (GSK3), and casein kinase 1α (CK1α). Within the destruction complex, β-catenin is phosphorylated by CK1α and GSK3 for ubiquitination and proteasomal degradation. Primary phosphorylation at Ser45 of β-catenin by CK1α is a prerequisite for GSK3-mediated phosphorylation at Thr41, Ser37, and Ser33, which is a pivotal event in the regulation of β-catenin in the cytosol [4]. In the existence of Wnt ligand, Wnt binds to its receptor complex and then the destruction complex is disturbed. This causes accumulation in the cytosol and nuclear translocation of β-catenin. A β-catenin/T-cell factor (TCF) transcription factor complex gives rise to the expression of target genes including c-Myc or cyclin D1, leading to the proliferation and metastasis of cancer cells [5]. Therefore, modulation of β-catenin levels in the aberrant activation of Wnt signaling is a potential therapeutic strategy for the chemoprevention and treatment of colon cancer.

Garlic (*Allium sativum* L., Amaryllidaceae) is one of the most important spices from ancient times and has been intensively studied for therapeutic properties, such as antithrombotic [6], lipid-lowering [7], anti-oxidative [8], and anti-hypercholesterolemia activities [9]. Ajoene, a mixture of *E*- and *Z*-isomers (*E*- and *Z*-4, 5, 9-trithiadodeca- 1, 6, 11-triene 9-oxide) is found in crushed garlic and has a broad variety of biological activities. Previously, we reported the anti-inflammatory [10], anti-oxidant [11], and anti-fatty liver activities of ajoene [12]. In this study, we report the modulatory activity of β-catenin by Z-ajoene from garlic as a regulator of the Wnt pathway and reveal its underlying mechanism.

## 2. Results and Discussion

The Wnt/β-catenin pathway has taken center stage in regulating embryonic development, tissue maintenance, and cell proliferation. In normal circumstances, the Wnt/β-catenin pathway is very elaborately controlled and β-catenin is conserved at a low level by way of ubiquitin-proteasomal degradation. However, deregulation of this pathway activates the transcription of β-catenin target genes, resulting in carcinogenesis and development of cancer, including colon cancer [13].

**Figure 1:**
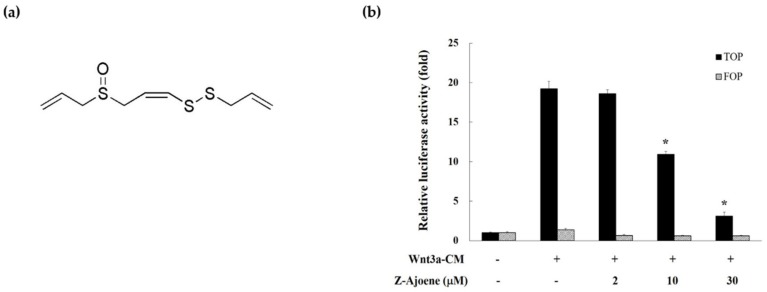
Effects of Z-ajoene on β-catenin response transcriptional activity in Wnt3a-activated HEK293 reporter cells. (**a**) chemical structure of Z-ajoene; (**b**) HEK293 reporter (TOPFlash) cells and control (FOPFlash) cells were incubated with Z-ajoene in the presence of Wnt3a-CM. After 15 h, luciferase activity was determined and expressed as a relative TOPFlash activity compared to vehicle-treated cells. The values are presented as the mean ± S.D. of three independent experiments. * *p* < 0.05 indicates a significant difference compared to Wnt3a-CM alone.

We measured β-catenin/TCF activity by evaluating the TOPFlash reporter activity in HEK293 reporter cells to examine the effect of Z-ajoene on transcriptional activity of β-catenin. Z-Ajoene is a chemically stable sulfur compound purified from crushed garlic (Figure 1a). We used Wnt3a-CM as a Wnt ligand to activate the Wnt/β-catenin pathway, showing high levels of TOPFlash reporter activity (Figure 1b). However, Z-ajoene concentration dependently repressed the Wnt3a-CM induced β-catenin related transcriptional activity. FOPFlash activity was used as a control reporter activity that was not affected by Z-ajoene and/or Wnt3a-CM treatment (Figure 1b). The results indicated that Z-ajoene can exert anti-cancer activity against cancer with a highly activated Wnt pathway. 

Colon cancer with mutations in APC is characterized by an abnormally activated Wnt pathway, which accounts for 75% of the causes of colon cancer. APC mutation results in the inactivated destruction complex for β-catenin degradation, β-catenin accumulation in the cytosol, and transcriptional activation of oncogenes including c-Myc and cyclin D1. Therefore, the Wnt/β-catenin pathway is a promising therapeutic target for colon cancer, although most therapeutic candidates are still at a very early developmental stage. Porcupine inhibitors, blockers of Wnt ligand secretion, impact tumors but not against APC truncated colon cancer since the loss of APC can activate the pathway without Wnt ligands. Moreover, tankyrase inhibitors target APC-mutated cancer by stabilizing axin; however, gastrointestinal toxicity has been reported [14].

**Figure 2:**
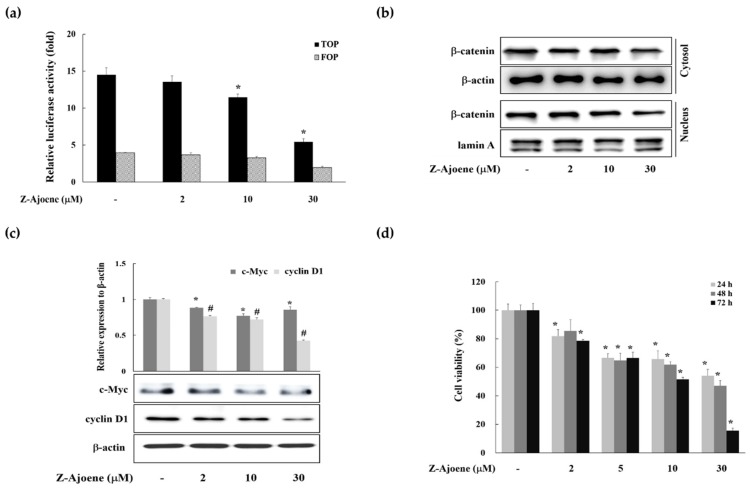
Effects of Z-ajoene on β-catenin response transcriptional activity, intracellular β-catenin levels, expressions of β-catenin target proteins, and cell proliferation in SW480 colon cancer cells. (**a**) SW480 cells were transfected with TOPFlash/FOPFlash and then incubated with Z-ajoene for 15 h. The luciferase activity is expressed as relative TOPFlash activity compared to vehicle-treated cells. * *p* < 0.05 indicates a significant difference compared to vehicle treatment; (**b**) the levels of β-catenin in the cytosol and nucleus were detected by Western blotting in SW480 colon cancer cells treated with Z-ajoene for 24 h; (**c**) SW480 cells were incubated with Z-ajoene for 24 h and cell lysates were prepared. Then, c-Myc and cyclin D1 were determined by Western blotting. The ratio of c-Myc (*) or cyclin D1 (#) to β-actin was expressed as relative to the vehicle-treated cells. *, # *p* < 0.05 indicates a significant difference compared to vehicle treated group. The images are representative of three independent experiments with similar results; (**d**) SW480 colon cancer cells were treated with various concentrations of Z-ajoene for 24, 48, and 72 h and cell viability was determined by the MTT assay. The values are presented as the mean ± S.D. of three independent experiments. * *p* < 0.05 indicates a significant difference compared to vehicle treatment.

To evaluate the anti-cancer activity of Z-ajoene, we used SW 480 colon cancer cells in which APC was truncated and showed constitutively high Wnt/β-catenin signaling without a ligand [15]. We observed the efficacy of Z-ajoene on β-catenin related transcriptional activity in SW480 colon cancer cells that were transiently transfected with a TOPFlash plasmid. As shown in Figure 2a, Z-ajoene repressed β-catenin/TCF activity in a concentration dependent manner. Z-Ajoene inhibited the levels of cytosolic and nuclear β-catenin in SW480 cells, and also inhibited protein expression of c-Myc and cyclin D1, although the effect on c-Myc was not concentration dependent (Figure 2b,c). Furthermore, Z-ajoene decreased SW480 cancer cell proliferation in a concentration-dependent manner (Figure 2d). These results indicated that Z-ajoene suppressed cell proliferation by downregulating β-catenin levels and the expression of β-catenin target proteins in SW480 colon cancer cells. 

**Figure 3:**
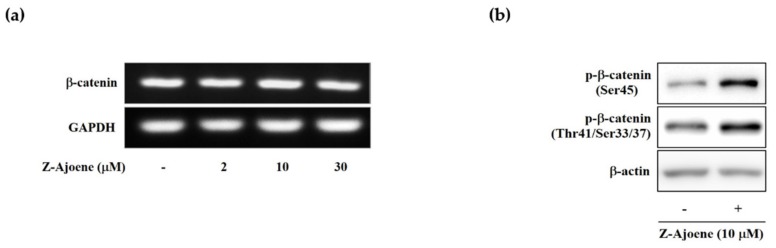
Effects of Z-ajoene on phosphorylation at Ser45 of β-catenin in SW480 colon cancer cells. (**a**) the mRNA levels of β-catenin were measured by RT-PCR in SW480 colon cancer cells with Z-ajoene for 15 h; (**b**) SW480 cells were treated with/without Z-ajoene in the presence of MG132 (proteasome inhibitor, 10 μM) for 15 h and then levels of phosphorylated β-catenin (p-β-catenin) were measured by Western blotting. The images are representative of three independent experiments with similar results.

To look into the regulatory mechanism of the downregulation of intracellular β-catenin levels by Z-ajoene, we measured the mRNA level of β-catenin by RT-PCR in SW480 colon cancer cells. Unlike the protein level, the mRNA expression of β-catenin was not affected by Z-ajoene (Figure 3a), indicating that Z-ajoene modulates β-catenin at a post-transcriptional step. Thus, we hypothesized that Z-ajoene could have an effect on phosphorylation and degradation of β-catenin without the aid of the destruction complex in the cytosol because truncated APC negates the destruction complex of β-catenin in SW480 cells. Z-Ajoene enhanced phosphorylation levels at Ser45 and Thr41/Ser37/33 of β-catenin in SW480 colon cancer cells (Figure 3b). Specifically, Z-ajoene dramatically increased the phosphorylation of β-catenin at Ser45, which is essential for the subsequent phosphorylation at Thr41/Ser37/33 and ubiquitin-dependent degradation [4]. These findings indicate that the phosphorylation of β-catenin at Ser45 is critical for the Z-ajoene-induced decrease in intracellular β-catenin in APC mutated SW480 colon cancer cells. 

**Figure 4:**
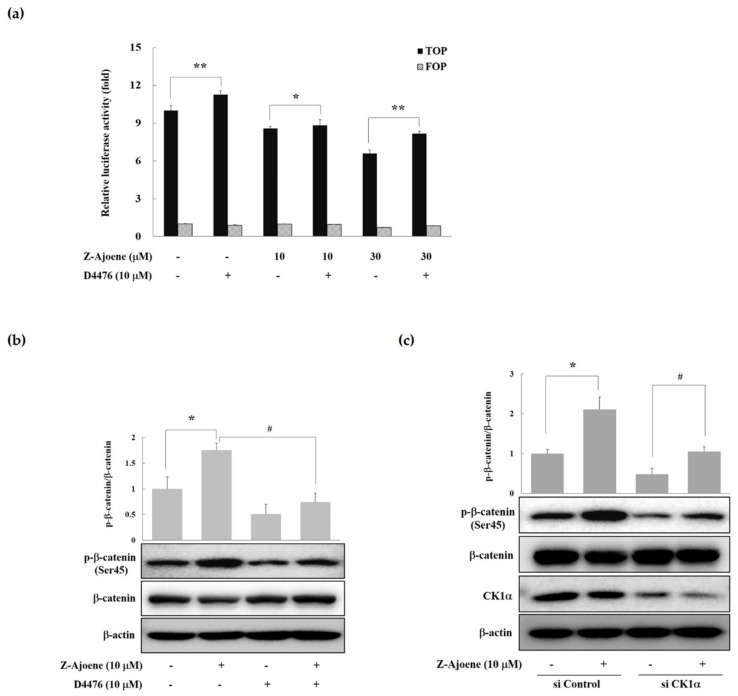
Effects of Z-ajoene and a CK inhibitor on phosphorylation at Ser45 of β-catenin in SW480 colon cancer cells. (**a**) SW480 colon cancer cells transfected with TOPFlash/FOPFlash were incubated with Z-ajoene and/or D4476 for 15 h. The luciferase activity was expressed as relative TOPFlash activity compared to vehicle-treated cells. The results represent the mean ± S.D. of three independent experiments. * *p* < 0.5 indicates a significant difference compared to Z-ajoene (10 μM) alone. ** *p* < 0.05 indicates a significant difference compared to vehicle or Z-ajoene (30 μM) alone; (**b**) the levels of phosphorylated β-catenin at Ser45 (p-β-catenin at Ser45) were evaluated by Western blotting. SW480 colon cancer cells were pre-treated with D4476 (a casein kinase I inhibitor, 10 µM), and then incubated with Z-ajoene for 30 min. The ratio of p-β-catenin (Ser45) to β-catenin was expressed relative to the values in the vehicle-treated cells. *, # *p* < 0.05 indicates a significant difference compared to vehicle (*) or a Z-ajoene (#) treated group. The images are representative of three independent experiments with similar results. (**c**) The levels of phosphorylated β-catenin at Ser45 (p-β-catenin at Ser45) were evaluated by Western blotting. SW480 colon cancer cells were transfected with control siRNA or CK1α siRNA (50 pM) and then incubated with Z-ajoene for 30 min. The ratio of p-β-catenin (Ser45) to β-catenin was expressed relative to the values in vehicle-treated cells. *, # *p* < 0.05 indicates a significant difference from vehicle treatment. The images are representative of three independent experiments with similar results.

CK1α, one of the CK1 isoforms (α, δ, γ1, γ2, and γ3) is responsible for the phosphorylation of β-catenin at Ser45 [16]. Therefore, we examined the effects of a CK1 inhibitor (D4476) on β-catenin/TCF activity. As seen in Figure 4a, treatment with D4476 reversed the Z-ajoene-induced downregulation of β-catenin-dependent transcriptional activities in SW480 colon cancer cells. These results suggest that Z-ajoene induced the degradation of intracellular β-catenin in a CK1α dependent manner. Z-Ajoene increased phosphorylated β-catenin at Ser45, which was reversed by D4476 in SW480 colon cancer cells (Figure 4b). We further confirmed the involvement of CK1α in the Z-ajoene-induced phosphorylation and degradation of β-catenin by using CK1α siRNA in SW480 colon cancer cells. Z-Ajoene-induced phosphorylation at Ser45 were decreased by treatment with CK1α siRNA, along with parallel decreases in the levels of CK1α protein (Figure 4c). Taken together, these results indicate that Z-ajoene reduced intracellular levels of β-catenin through the amplification of β-catenin phosphorylation at Ser45 in a CK1α-dependent manner in SW480 colon cancer cells. Pyrvinium, a new class of Wnt inhibitors, potentiated CK1α kinase activity and decreased colon cancer cell viability [17]. Pyrvinium treatment also suppressed nuclear β-catenin levels in the intestines of APCmin mice [18]. Here, we firstly report that Z-ajoene, a compound from natural product, increased β-catenin phosphorylation in a CK1α-dependent manner. In addition, this finding suggests that CK1α could be a target of Z-ajoene to regulate APC mutant colon cancer. The detailed mechanism of CK1α activation by Z-ajoene should be further investigated. 

We confirmed the inhibitory effect of Z-ajoene on the Wnt/β-catenin signaling pathway using a Wnt3a-CM-activated HEK293 reporter cell system. Z-Ajoene repressed intracellular levels of β-catenin without changing the transcriptional expression of β-catenin. In addition, Z-ajoene suppressed TOPFlash reporter activity in LiCl (GSK3β inhibitor) activated HEK293 reporter cells, which means that inhibitory activity of Z-ajoene on Wnt/β-catenin signaling pathway is not involved in GSK3β activity. Consistent with the effects of Z-ajoene in SW480 colon cancer cells, phosphorylated β-catenin was increased by Z-ajoene. A CK1 inhibitor reversed the effect of Z-ajoene treatment in HEK293 reporter cells by increasing β-catenin/TCF activity. These results suggest that Z-ajoene works as an inhibitor of the Wnt/β-catenin signaling pathway by the amplification of CK1α-dependent β-catenin phosphorylation (Supplementary Materials, Figure S1).

Taken together, the results indicate that Z-ajoene inhibited the Wnt/β-catenin signaling pathway by downregulating intracellular β-catenin levels through the promotion of CK1α-dependent phosphorylation of β-catenin, resulting in the suppression of SW480 colon cancer cell growth.

## 3. Materials and Methods

### 3.1. Preparation of Z-Ajoene from Crushed Garlic Extracts

Z-Ajoene was isolated and the structure was elucidated as described previously [10].

### 3.2. Preparation of Z-Ajoene from Crushed Garlic Extracts Cell Lines, Transfection, and Luciferase Assay

SW480 cells, human colorectal adenocarcinoma cells with truncated APC, was obtained from the Korea Cell Line Bank (Seoul, Korea) and cultured in DMEM with 10% fetal bovine serum (FBS). HEK293 reporter cells (stably transfected with TOPFlash, a synthetic β-catenin/TCF-dependent luciferase reporter and human Frizzled-1 expression plasmids) and control cells (stably transfected with FOPFlash, a negative control reporter with mutated β-catenin/TCF binding elements) were kindly provided by Sangtaek Oh (Kookmin University, Seoul, Korea). Wnt3a-secreting L cells (L-Wnt3a cells) were obtained from the American Type Culture Collection (Manassas, VA, USA). The TOPFlash reporter plasmid was transfected into SW480 cells using Lipofectamine PLUS reagent (Life Technologies, Carlsbad, CA, USA) according to the manufacturer’s instructions. After 24 h transfections, cells were treated with Z-ajoene for 15 h. The luciferase activity of the SW480 cell lysates was measured by a luminometer (Molecular Devices, Sunnyvale, CA, USA).

### 3.3. Preparation of Wnt3a-Conditioned Medium (Wnt3a-CM)

Wnt3a-CM was prepared according to the manufacturer’s instruction. Wnt3a-secreting L cells were cultured in DMEM with 10% fetal bovine serum (FBS) for four days. The medium was harvested and followed by sterilization using a 0.22-μm filter. Fresh medium was added to the cells and cultured for another three days. The collected medium was combined with the previous medium.

### 3.4. Reporter Gene Assay

The HEK293 reporter cells (with TOPFlash reporter plasmid) or control cells (with FOPFlash reporter plasmid) were inoculated at 1000 cells/well and grown for 24 h. After 15 h incubation with Z-ajoene and/or Wnt3a-CM, luciferase activity in the cell lysates was measured by a luciferase assay system (Promega, Madison, WI, USA).

### 3.5. Cell Viability

Cells (1.5 × 10^3^ cells/well) were treated with Z-ajoene (various concentrations) for 24, 48, and 72 h, and the cell viability was measured in triplicate using a 3-(4,5-dimethylthiazol-2-yl)-2,5-diphenyl tetrazolium bromide (MTT) method. 

### 3.6. Western Immunoblots

For preparation of whole-cell lysis extracts, the cells were treated with Z-ajoene for 24 h and lysed with cell lysis buffer (Cell Signaling Technologies, Beverly, MA, USA). The cell lysates were then centrifuged at 10,000× *g* for 20 min at 4 °C. The supernatants were collected and protein concentrations were assessed using a BCA protein assay kit. The cytosolic and nuclear fractions were prepared as previously described with some adjustment [19]. Proteins were separated on 10% sodium dodecyl sulfate-polyacrylamide gel electrophoresis (SDS-PAGE) and transferred to polyvinylidene fluoride (PVDF) membranes (GE Healthcare, Piscataway, NJ, USA). The membranes were probed with antibodies and visualized using the ECL chemiluminescence (GE Healthcare). The antibodies against β-catenin (BD Transduction Laboratories, San Jose, CA, USA), c-Myc, β-actin, lamin A (Cell Signaling Technology, Beverly, MA, USA) and cyclin D1 (Santa Cruz Biotechnology, Dallas, TX, USA) were used to detect the target proteins.

### 3.7. RT-PCR Analysis

Cells (1 × 10^6^ cells/dish) were treated for 15 h with/without Z-ajoene. Total RNA was isolated using RNA-isolation Trizol reagent (Life Technologies, Carlsbad, CA, USA) and then reverse transcribed into cDNA using reverse transcriptase and random hexamer. The cDNA was mixed with buffer, dNTP, Taq DNA polymerase (Promega, Madison, WI, USA) and primers. The sense and antisense primers for β-catenin were 5′-GGGATGTTCACAACCGAATTGTTATC-3′ and 5′-ACCAGAGTGAAAAGAACGATAGCTAGGA-3′, respectively. The sense and antisense primers for β-actin were 5′-TGTGATGGTGGGAATGGGTCAG-3′ and 5′-TTTGATGTCACGCACGATTTCC-3′, respectively. The amplified DNA was separated on 1.5 % agarose gels and stained with ethidium bromide.

### 3.8. Statistics

The data were expressed as the mean ± S.D. of three experiments, and statistical analysis was performed by the Student’s t-test. A *p*-value of < 0.05 was considered to indicate a significant difference.

## Supplementary Materials

The following are available online at https://www.mdpi.com/1420-3049/25/3/703/s1, Figure S1: Effects of Z-ajoene on Wnt/β-catenin pathway in Wnt3a-activated HEK293 reporter cells.
