# Supplementary material for: Z-Ajoene Inhibits Growth of Colon Cancer by Promotion of CK1α Dependent β-Catenin Phosphorylation
Source: Molecules. 2020 Feb 6;25(3):703. doi: 10.3390/molecules25030703 (PMC7037095; doi:10.3390/molecules25030703)
Supplement: Supplementary file 1 [file molecules-25-00703-s001.zip › revised Supplementary figure caption_Fig.S1-Ryu.pdf]

Figure caption (Supplementary material, Figure S1)

Figure S1. Effects of Z-ajoene on Wnt/β-catenin pathway in Wnt3a-activated HEK293 reporter cells. (**a**) The levels of β-catenin were detected by Western blotting in HEK293 reporter (TOPFlash) cells treated with Z-ajoene in the presence of Wnt3a-CM for 15 h. Images are representatives of three independent experiments with similar results. (**b**) mRNA levels of β-catenin were measured by RT-PCR in HEK293 reporter (TOPFlash) cells treated with Z-ajoene in the presence of Wnt3a-CM for 15 h. (**c**) HEK293 reporter (TOPFlash) cells and control (FOPFlash) cells were incubated with Z-ajoene in the presence of LiCl (GSK3β inhibitor). After 15 h incubation, luciferase activity was determined and expressed as relative TOPFlash activity compared to vehicle-treated cells. The values are presented as the mean ± S.D. of three independent experiments. *$p < 0.05$ indicates a significant difference compared to LiCl alone. (**d**) The levels of phosphorylated β-catenin (p-β-catenin) were assessed in HEK293 reporter (TOPFlash) cells treated with Z-ajoene (10 μM) in the presence of MG132 (proteasome inhibitor, 10 μM) and/or Wnt3a-CM for 15 h. Images are representatives of three independent experiments with similar results. (**e**) HEK293 reporter (TOPFlash) cells and control (FOPFlash) cells were pre-treated with D4476 for 1 h, then incubated with Z-ajoene in the presence of Wnt3a-CM for 15 h. The relative luciferase activity was expressed as the relative TOPFlash activity compared to vehicle treated cells. The values are presented as the mean ± S.D. of three independent experiments. *$p<0.05$ indicates a significant difference from Z-ajoene treatment in presence of Wnt3a-CM.
