# Supplementary figures and images for: Z-Ajoene Inhibits Growth of Colon Cancer by Promotion of CK1α Dependent β-Catenin Phosphorylation
Source: Molecules. 2020 Feb 6;25(3):703. doi: 10.3390/molecules25030703 (PMC7037095; doi:10.3390/molecules25030703)

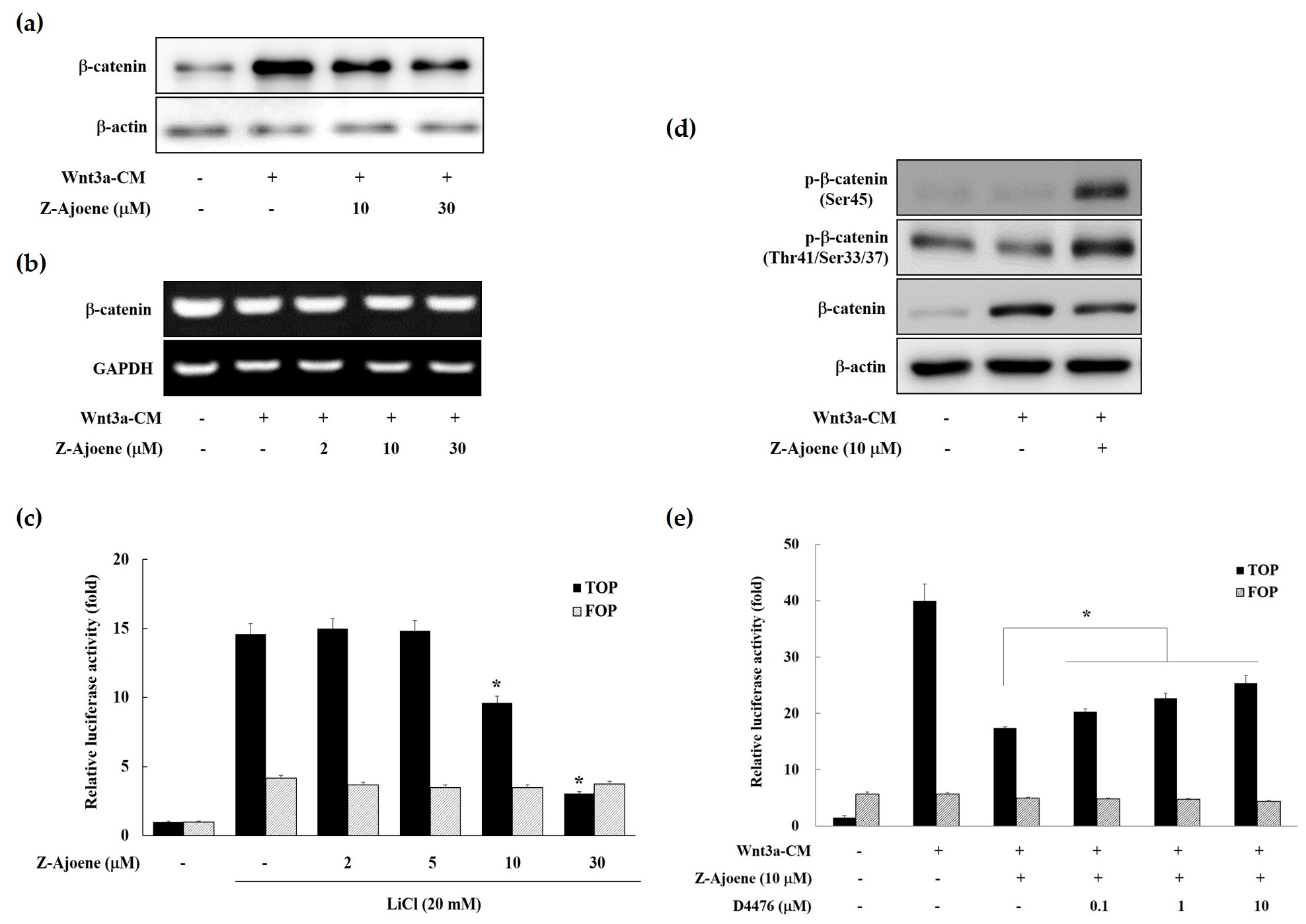

Supplement: Supplementary file 1 [file molecules-25-00703-s001.zip › revised Supplementary Fig.S1-Ryu.tif]
